# Humidity Sensor Composed of Laser-Induced Graphene Electrode and Graphene Oxide for Monitoring Respiration and Skin Moisture

**DOI:** 10.3390/s23156784

**Published:** 2023-07-29

**Authors:** Xianxiang Fei, Junyi Huang, Wenqing Shi

**Affiliations:** 1 School of Electronics and Information Engineering, Guangdong Ocean University, Zhanjiang 524088, China; swqafj@126.com; 2 College of Mechanical Engineering, Guangdong Ocean University, Zhanjiang 524088, China; m13660603311@163.com

**Keywords:** laser-induced graphene, humidity sensors, respiration monitoring, graphene oxide, interdigital electrode

## Abstract

Respiratory rate and skin humidity are important physiological signals and have become an important basis for disease diagnosis, and they can be monitored by humidity sensors. However, it is difficult to employ high-quality humidity sensors on a broad scale due to their high cost and complex fabrication. Here, we propose a reliable, convenient, and efficient method to mass-produce humidity sensors. A capacitive humidity sensor is obtained by ablating a polyimide (PI) film with a picosecond laser to produce an interdigital electrode (IDE), followed by drop-casting graphene oxide (GO) as a moisture-sensitive material on the electrode. The sensor has long-time stability, a wide relative humidity (RH) detection range from 10% to 90%, and high sensitivity (3862 pF/%RH). In comparison to previous methods, the technology avoids the complex procedures and expensive costs of conventional interdigital electrode preparation. Furthermore, we discuss the effects of the electrode gap size and the amount of graphene oxide on humidity sensor performance, analyze the humidity sensing mechanism by impedance spectrum, and finally perform the monitoring of human respiratory rate and skin humidity change in a non-contact manner.

## 1. Introduction

Humidity sensors have a large number of applications in many fields, such as agricultural production, industrial manufacturing, food processing, and environmental monitoring [1,2,3,4]. Humidity sensors have recently been applied to monitor human health [5,6]. With the global spread of the COVID-19 virus, non-contact sensing and respiratory monitoring have become important tools to prevent and control respiratory infectious diseases. The moisture content in breath and skin moisture change can reflect the body’s metabolism and health status, so it is especially important to obtain data on changes in respiratory rate and human skin humidity by a non-contact method, which poses a great challenge to the sensitivity, real-time and reliability of humidity sensor.

There are many kinds of humidity sensors, mainly capacitance [7,8], impedance [9,10], current [11], voltage (i.e., self-powered humidity sensors) [12,13,14], fiber optic [15], quartz crystal microbalance (QCM) [16], and resonant surface acoustic wave (SAW) [17]. In terms of power supply systems, humidity sensors can be classified into passive sensors and self-powered sensors. Passive sensors are limited in their application due to the need for an external power supply. Compared to traditional passive sensors, self-powered sensors are of interest because they can be powered. However, during the process of power supply, a redox reaction occurs [14], which reduces the lifetime of the sensor, and the energy storage problem of self-powered sensors is also to be solved. Additionally, humidity sensors can be classified into wired and wireless sensors [18]. How to transmit signals without power and wires is a direction of research. Although people put forward higher requirements for humidity sensors, the requirements of low cost and high accuracy for sensors are always the same. We have made some useful explorations on the above two aspects in this paper. Most humidity sensors are composed of humidity-sensitive materials and electrodes. A variety of humidity-sensitive materials have been developed, including metal oxides (e.g., titanium dioxide [19] tricobalt tetroxide [20]), ceramics (e.g., halloysite nanotubes [21], attapulgite [22], sepiolite nanofibers [23]), cellulose paper [24], polymers [25], MXene [26] and carbon materials [27] (e.g., graphene [28], graphene oxide (GO) [29], and carbon nanotubes [30]), among which graphene oxide is of interest for its excellent humidity sensing capability. Graphene oxide is a two-dimensional graphene derivative with a high specific surface area. Its surface and edges are covered with a large number of epoxy, hydroxyl, and carboxyl groups. These functional groups are hydrophilic [31,32,33,34], making GO suitable as a humidity-sensitive material. For example, Li et al. coated Nafion and graphene oxide quantum dot (GOQD) composites on the electrode to fabricate a capacitive humidity sensor, and the addition of GOQD resulted in a faster response time [35]. Guo et al. greatly improved the sensitivity of the sensor by using MoS_2_-modified graphene oxide on Au electrodes as a sensitive film [36]. The improvement of sensor performance is mainly via the modification of the sensing layer, which necessarily results in a more complex preparation process and device structure. On the other hand, conventional electrodes are typically pricey metal electrodes (gold, silver, platinum, copper, etc.) with limited reserves [37,38,39], and the cost of electrodes is higher. Recently, laser-induced graphene (LIG) technology has received increasing attention [40,41], which forms a carbon electrode on the polymer surface in an ambient atmosphere by laser direct writing. When the laser energy is high enough, sp^3^-hybridized carbon atoms experience photothermal conversion to sp^2^-hybridized carbon atoms, and the polymer is converted into a conductive graphene structure. The approach not only reduces the cost of electrodes, but is non-toxic, photomask-free, and has a controllable electrode shape, which has the potential for large-scale applications.

In this paper, a picosecond laser is utilized to ablate polyimide (PI) film to form a conductive interdigital electrode (IDE), and then GO solution is coated onto the interdigital electrode to create a humidity-sensitive layer. This method not only reduces the cost of the sensor but also simplifies the process. The humidity is monitored by measuring the capacitance of the sensor. The sensor has a high capacitive response over an RH range of 10% to 90%. The sensors also feature small humidity hysteresis and good stability. To obtain the best performance, sensors with different gap sizes of the interdigital electrode and different GO amounts are prepared for analysis. The excellent performance of the sensor is demonstrated by breathing monitoring and non-contact sensing of skin humidity.

## 2. Materials and Methods

### 2.1. Materials

Polyimide film was purchased from Xinhongsen Technology Co., Ltd. (Shenzhen, China). The conductive silver glue was obtained from Kaixiang Electronic Products Co., Ltd. (Guangzhou, China). Graphene oxide (2 mg/mL) was purchased from Guoheng Qihang Co., Ltd. (Shenzhen, China).

### 2.2. Fabrication of Humidity Sensor

**Figure 1:**
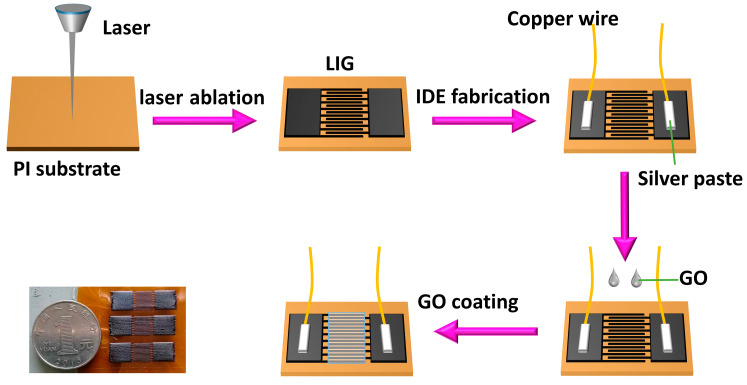
Schematic diagram of the sensor fabrication process.

As shown in Figure 1, PI film of 100 μm thickness was first cut into a square (25 mm × 25 mm) as a substrate and then taped to the glass sheet. Next, the interdigital electrodes were generated by ablating the PI sheet with a 1030 nm wavelength picosecond laser (IPG Photonics). The electrode had eight pairs of interdigital fingers with a finger length of 6 mm. The average power of the laser was 50 W, the power percentage was 20%, and the frequency was 200 kHz. Then, the copper wire was glued to the common electrode with conductive silver glue. Finally, the GO solution was drop-coated on the electrode with a pipetting gun. It was dried naturally in the room for 48 h to make a uniform GO film.

### 2.3. Materials Characterizations

A Leica DVM6 depth-of-field (DOF) microscope was used to observe the morphology of GO and LIG. Raman spectra were observed with a Raman spectroscopy (Pioneer Technology RTS2, Bordentown, NJ, USA). Raman excitation source was a laser of 532 nm. The sensor capacitance was measured using an LCR meter (TH2829A, Tonghui Electronic Co. Ltd., Changzhou, China). A high-precision Bluetooth humidity sensor (Jiali Technology Co., Ltd., Chengdu, China) was used to monitor humidity with a humidity accuracy of 1.5% RH and humidity resolution of 0.1% RH. The gas flow rate was controlled by a gas flow controller (LZB-3WB, Shunlaida Measurement Co., Ltd., Nanjing, China).

### 2.4. Humidity Sensing System

**Figure 2:**
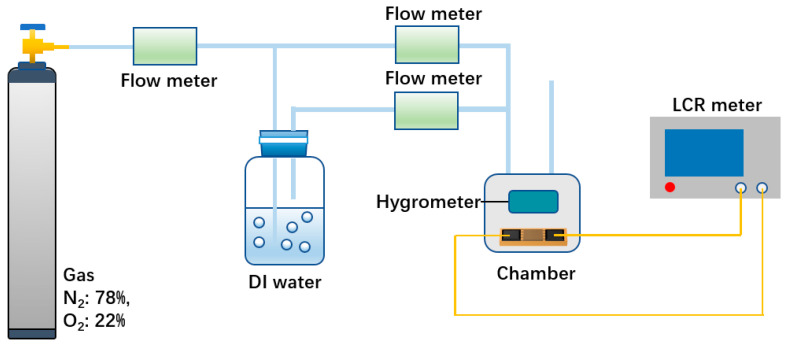
Schematic of the humidity measurement system.

As shown in Figure 2, all measurements were performed at 30 °C. The gas coming from the synthetic air (N_2_: 78%, O_2_: 22%) bottle was dry gas with a flow rate of 1 L/min, and the gas bubbling through the deionized water was moist gas. The dry gas was mixed with the moist gas in different proportions by adjusting the flow meter to obtain a stable humidity environment. We placed the sensor in the test chamber and measured its capacitance with LCR. The ambient humidity in the test chamber could be measured in real time by a commercial hygrometer. Humidity sensitivity is denoted by the formula S = $\frac{C_{RH}-C_{0}}{RH-{RH}_{0}}$, where S denotes the sensitivity, *C_RH_* denotes the capacitance at *RH*% humidity, and *C*_0_ denotes the capacitance at *RH*_0_% humidity. The response and recovery time of the sensor is the time it takes for the sensor capacitance to go from the initial value to 90% of the stable value.

## 3. Results and Discussion

### 3.1. Morphology and Structure Analysis of LIG

**Figure 3:**
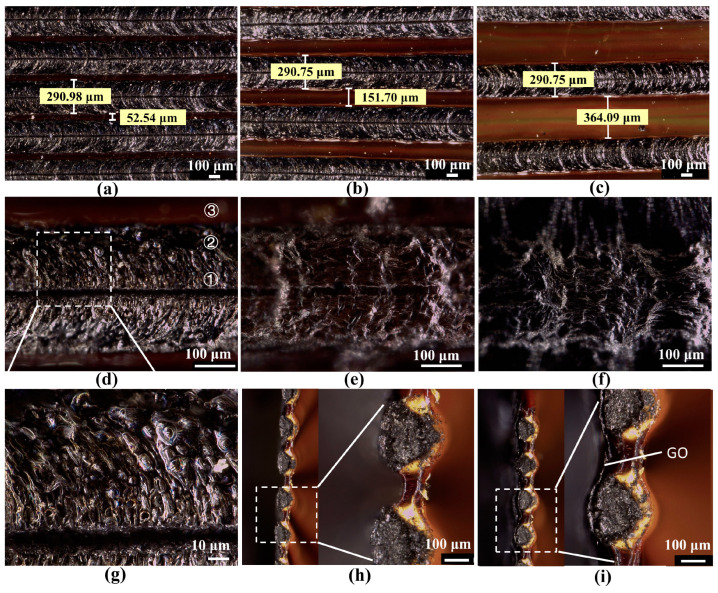
Morphology of the humidity sensors. (**a**–**c**) Depth of field (DOF) images of PI-based sensors with different electrode gap sizes, (**a**) 50 μm gap, (**b**) 150 μm gap, and (**c**) 360 μm gap. (def) DOF images of GO-based humidity sensors with drop-coated, (**d**) 0 μL GO solutions, I 30 μL GO solutions, and (**f**) 120 μL GO solutions. (**g**) Enlarged 3D DOF image. (**h**) DOF image of the PI-based sensor’s cross-section. (**i**) DOF image of the GO-based sensor’s cross-section.

Three sensors with different electrode gaps are designed, 50 μm, 150 μm, and 360 μm corresponding to electrode areas of 32.34 mm^2^, 41.34 mm^2^, and 59.34 mm^2^, respectively, with an electrode width of 290 μm, as shown in Figure 3a–c. Sensors that are not drop-coated with GO are called PI-based sensors. The sensor with GO drop-coated is called a GO-based sensor. For convenience, we named the sensors with different gap sizes and different volumes of GO solution as LIG_M_-N, where M represents the gap size, and N represents the volume of GO solution. For example, in LIG_150_-60, 150 represents the gap size of 150 μm, and 60 represents 60 μL of GO solution.

As shown in Figure 3d,g in the absence of GO, the surface is black with a gap in the middle and has more small holes with a diameter of about 3–5 μm. In the process of laser ablation, the laser is emitted at a certain frequency, resulting in a crumpled carbon electrode morphology with layers of stacking accompanied by bulges and small holes. Bulges are caused by PI melting, and holes are caused by PI bulge cracking. The middle gap is formed because the instantaneous power of the laser is so high that it raises the PI film to more than 1000 K, and an explosive phase change occurs, resulting in boiling, vaporization, and decomposition of the polyimide. The high temperature causes carbonization of the PI film, which results in microhumps around the ablation area. From the cross-sectional DOF image in Figure 3h, the carbon layer is about 50 μm higher than the PI film. Figure 3i shows that the PI film becomes a layered structure after drop-casting GO, and the whole electrode has three parts: the bottom layer of yellow PI, the middle layer of a black carbon electrode, and the upper layer of GO. When the GO solution is 30 μL, the GO film is flat, and the gap can be clearly seen, while when the GO solution is 120 μL, the gap becomes blurred, and the GO film is porous, as seen in Figure 3d–f. It confirms that the thickness of the GO layer becomes thicker as the amount of GO increases.

**Figure 4:**
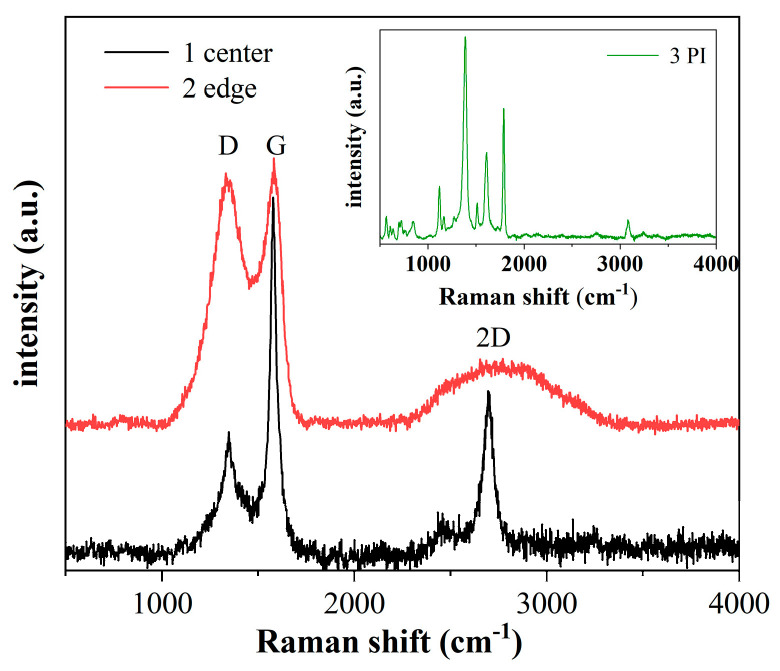
Raman spectra of LIG, The collection points of Raman spectroscopy are marked with corresponding numbered circles in Figure 3d, (1) near the electrode center, (2) about 100 μm from the electrode center, and (3) PI film, inset is Raman spectra of PI.

As shown in Figure 4, point (3) is obviously different from points (1) and (2), and point (3) is a typical polyimide characteristic peak [42]. In contrast, points (1) and (2) have distinct peaks characteristic of the carbon structure, i.e., D-peak, G-peak, and 2D-peak, corresponding to positions at 1357 cm^−1^, 1580 cm^−1^, and 2695 cm^−1^. These peaks are identified as a graphene structure, with the D peak representing the amorphous carbon structure and the G peak representing the carbon–carbon bond stretching, which can be considered as a graphite structure. The ratio of 2D to G can be a good indication of the presence of high-quality monolayer graphene. I_2D_/I_G_ is 0.5 near the center of the electrode, and it can be inferred that there is a multi-layer graphene with around 4–5 layers [43]. There is no obvious 2D peak in the electrode’s edge spectrum, but there are distinct D and G peaks. It indicates the formation of the graphite and amorphous carbon structure. Near the center, the temperature is quite high, and the polyimide decomposes fast, producing higher mass graphene, whereas the edge region has a lower temperature and slower decomposition, producing amorphous carbon and a thicker graphite structure. The presence of graphene enhances the conductivity of the electrode.

### 3.2. Humidity Sensing Characteristics

#### 3.2.1. Comparison of PI-Based Sensors with Different Electrode Gap Sizes

**Figure 5:**
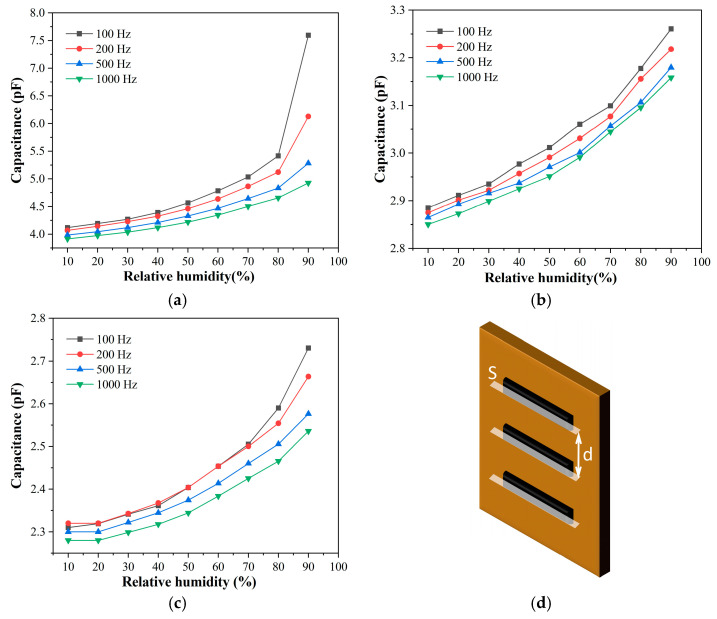
The capacitance of PI-based sensor varies with relative humidity at different frequencies, with different electrode gap sizes, (**a**) 50 μm gap, (**b**) 150 μm gap, and (**c**) 360 μm gap. (**d**) Schematic illustration of parallel-plate capacitor consisting of interdigital electrodes.

Figure 5a–c shows the humidity response performance of PI-based sensors with different gaps. The capacitance of the sensor decreases gradually with increasing gap size. This phenomenon can be qualitatively explained by the equation C = εε_0_S/d for parallel-plate capacitor, where d is the gap size, S is the cross-sectional area of the electrode finger, and ε is the relative permittivity of the interdigital electrodes, ε_0_ is the dielectric constant in vacuum, as shown in Figure 5d. Additionally, the capacitance of these sensors increases as the relative humidity rises in Figure 5a–c. ε_water_ is 78.4, which is much higher than 3.4 of ε_PI_ in the electrostatic field. When the humidity rises, the PI film absorbs more water molecules, increasing the dielectric constant of PI film and thus an increase in capacitance. The high dielectric constant of water is because water molecules are polarized in an electric field. This polarization can better respond to the low-frequency electric field. When the frequency of the electric field increases, the polarization speed of water molecules cannot keep up with the change in the direction of the electric field [44,45]. Therefore, the dielectric constant increases more slowly. The capacitive response of the sensor is maximum at 100 Hz. Although the humidity sensor based on PI responds to changes in humidity, its sensitivity is relatively low. Therefore, we enhance the sensitivity of the sensor by applying GO on the surface of the interdigital electrode.

#### 3.2.2. Comparison of GO-Based Sensors with Different Electrode Gap Sizes

**Figure 6:**
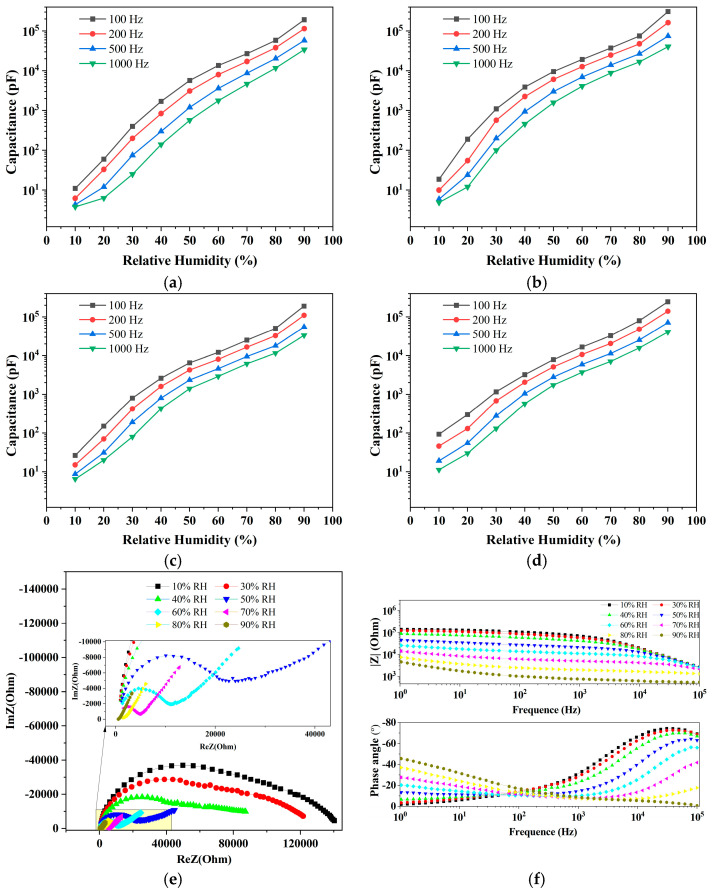

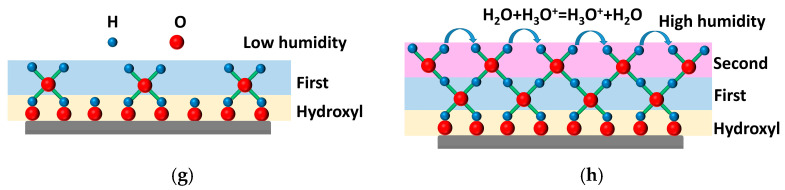
Capacitance vs. relative humidity for a GO-based sensor with a 150 μm gap, using (**a**) 30 μL GO solutions, (**b**) 60 μL GO solutions, (**c**) 90 μL GO solutions, and (**d**) 120 μL GO solutions. (**e**) Impedance spectroscopy of LIG_150_-60 measured at different RH levels, inset is enlarged curve. (**f**) Bode diagram of LIG_150_-60 at different RH levels. (**g**,**h**) Schematic illustration of the humidity sensing principle of LIG_150_-60, (**g**) low humidity less than 40% and (**h**) high humidity higher than 50%.

For the three gap sizes (50 μm, 150 μm, 360 μm) of GO-based sensors, the drop-coated GO solution is 30 μL, 60 μL, 90 μL, and 120 μL, among which the sensitivity of LIG_150_-60 is the highest, as seen in Table 1. Figure 6a–d depicts the capacitance response curves of the sensor with a gap size of 150 µm for various GO loadings. It is clear that the smaller the frequency, the higher the capacitance response, which is consistent with PI-based sensors. Therefore, we used a frequency of 100 Hz to evaluate the performance of the sensor. As shown in Figure 6b, when the humidity varies from 10% to 90% RH, the capacitance of LIG_150_-60 changes from 18.8 pF to 3.09 × 10^5^ pF. The sensitivity reaches 3862 pF/%RH, which is much greater than that of PI-based sensors. It is not sufficient to explain the change in capacitance by an increase in the dielectric constant between the interdigital electrodes. In addition, the capacitance response increased more significantly at high RH than at low RH. We can try to explain this phenomenon by impedance spectroscopy.

As seen in Figure 6e of the LIG_150_-60 impedance spectrum, at 10% to 30% humidity, it is an approximate semicircle; the diameter of the semicircle can be used as the internal resistance of the sensor [46], about 100 KΩ to 140 KΩ. Due to a large number of hydrophilic groups on the GO surface [47], water molecules are mostly chemisorbed on the GO surface by hydrogen bonding and cannot move freely. As shown in Figure 6g, the conductivity mainly depends on electrons in the electrode and GO. The increase in capacitance relies mainly on the increase in dielectric constant after the adsorption of water. As the humidity continues to increase, the first water molecules layer is formed. When the humidity rises to 50%, the diameter of the semicircle continues to decrease, meaning that the internal resistance of the sensor decreases, while a straight line with an approximate 45-degree slope appears in the low-frequency band, indicating the appearance of Warburg impedance, which is caused by the diffusion process of charge carriers at the GO film/electrode interface. At this point, due to the increasing number of water molecules, a physical adsorption layer is formed on the first water molecules layer, as illustrated in Figure 6h. According to the Grotthuss transport mechanism (H_2_O + H_3_O^+^ = H_3_O^+^ + H_2_O), [21,48], hydrated hydrogen ions are formed in the adsorbed layer as conductive carriers, and the capacitance of the sensors depends mainly on the diffusion of hydrated hydrogen ions at the GO membrane/electrode interface. At humidity greater than 80%, multiple layers of physical adsorption have been formed, at which point the semicircle has disappeared, and there is only a straight line, indicating that the sensor performance is mainly determined by the Warburg impedance generated by ion diffusion. The higher the humidity, the more hydrated hydrogen ions are present, and the diffusion capacitance increases sharply.

Figure 6f shows the bode diagram of LIG_150_-60 at different humidity levels. From the phase diagram, it can be seen that the phase curves at all humidity levels basically intersect at the position of 100 Hz, which indicates that the phase angle is basically unchanged at 100 Hz regardless of the humidity level. Therefore, the change in impedance at 100 Hz can represent the change in capacitance. At other frequencies, the impedance and phase angle vary with humidity, and the uncertainty increases. The selection of 100 Hz frequency as the test frequency also considers this factor. As can be seen from the impedance diagram in Figure 6f, at 100 Hz, as humidity rises, the impedance also becomes gradually smaller, representing a gradual increase in capacitance, a result that is also consistent with Figure 6b.

**Figure 7:**
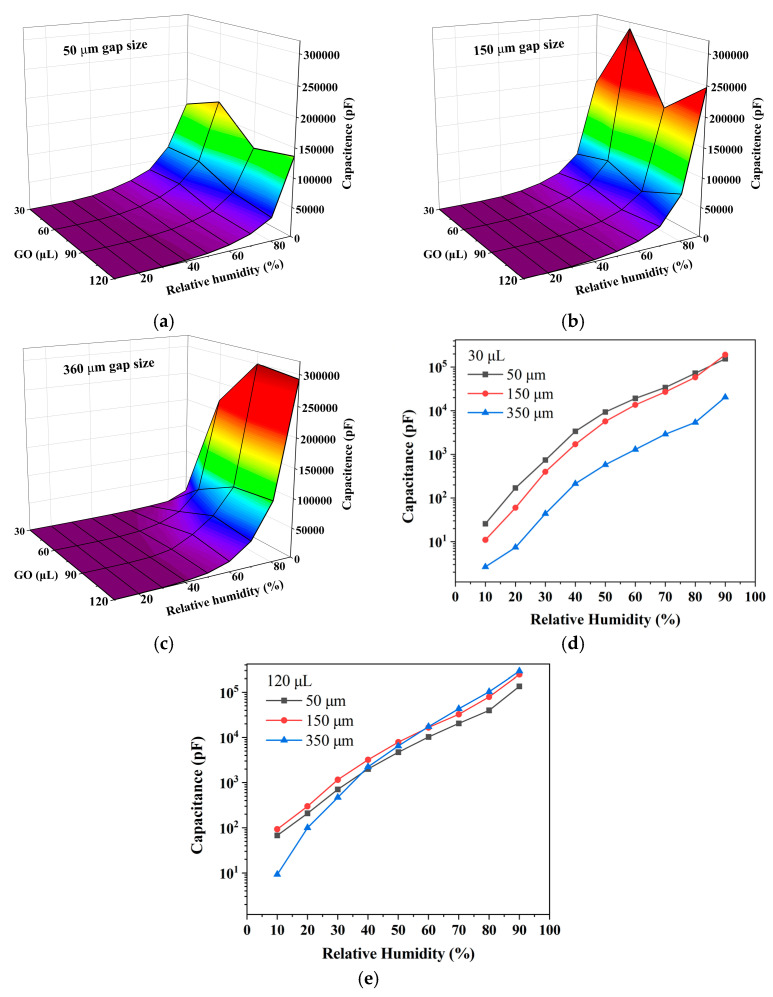
The capacitance of sensors with different GO loadings varies with relative humidity at 100 Hz, (**a**) gap size of 50 μm, (**b**) gap size of 150 μm, and (**c**) gap size of 360 μm. (**d**,**e**) The capacitance of GO-based sensors with different gaps varies with relative humidity at different GO loadings of (**d**) 30 μL and (**e**) 120 μL.

To investigate the effect of different gaps and different GO amounts on the performance of the humidity sensor, we measured the capacitive response of the sensor with different parameters, as shown in Figure 7a–c. Figure 7a shows that the sensitivity of the sensor is not monotonically increasing with the amount of GO but has an optimal value. When the GO solution is 60 μL, the capacitive response is the largest, and at 90 μL, the capacitive response becomes smaller instead. This may be because the thickness of GO film increases as the amount of GO increases, resulting in a larger resistance between the carbonized electrode and GO film, making the capacitive response smaller. Figure 7b is similar, and Figure 7c is slightly different, mainly because the area of the electrode with a 360 μm gap size is almost twice as large as that with a 50 μm gap size, resulting in less GO per unit area. Based on the electrode area and the amount of GO, it can be obtained that the capacitive response is better when the amount of GO is 1.45–1.86 μL/mm^2^. The details are shown in Table 1.

Figure 7a–c further shows that the sensor with a 50 μm gap is not the most sensitive; rather, the sensor with a wider gap is more sensitive. The sensor with a narrow gap has a limited area, and when the GO solution is 30 μL, there are fewer hydrated hydrogen ions at high relative humidity, resulting in a weak diffusion capacitance. As shown in Figure 7d, the capacitive response is inversely proportional to the gap size at this point. Additionally, when the GO solution is 120 μL, the electrodes with larger gaps produce more hydrated hydrogen ions due to their larger areas, the ion diffusion is enhanced, and the diffusion capacitance grows rapidly at high relative humidity. As seen in Figure 7e, the capacitive response at this time is positively correlated with the electrode gap. In summary, when the amount of GO is little, the sensitivity is inversely related to the electrode gap size, while when the amount of GO is sufficient, the sensitivity is proportional to the electrode gap.

#### 3.2.3. Sensor Response Time and Stability

**Figure 8:**
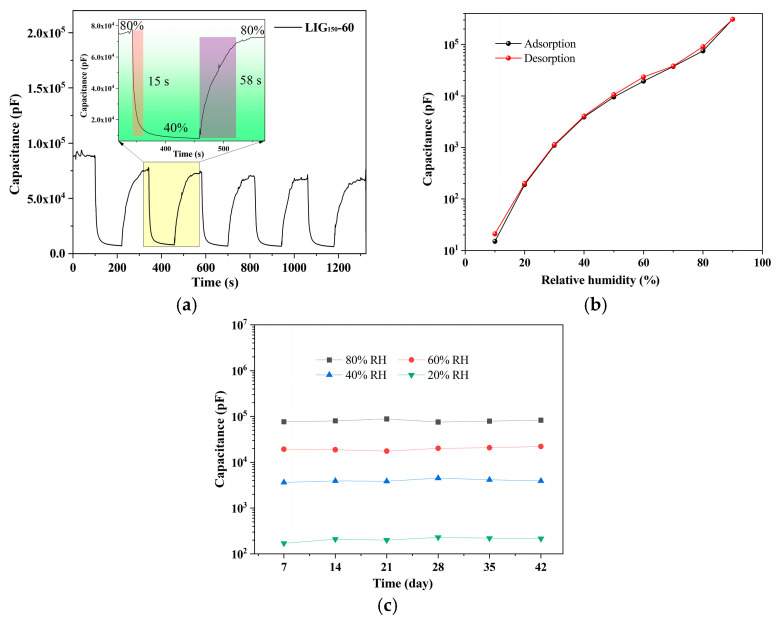
(**a**) Dynamic capacitance response curves from 40% to 80% RH for five cycles, and the inset is the dynamic response and recovery time for the LIG_150_-60 sensor. (**b**) Hysteresis curves for the LIG_150_-60 sensor. (**c**) The LIG_150_-60 sensor’s long-term stability in environments with a relative humidity of 20%, 40%, 60%, and 80%.

Figure 8a shows the capacitive response of LIG_150_-60 experiencing five cycles between 80% and 40% RH, and it can be seen that the adsorption time is 58 s and the desorption time is 15 s. As mentioned above, water molecules will combine with hydrophilic groups and adsorb on the GO surface when the humidity rises. However, the adsorption process is not uniform, and there may be some areas where more water molecules have been adsorbed, while others may not have water molecules yet, which results in the ion diffusion process being hindered. The first layer of chemisorption must be completed on the GO surface, and the Grotthuss effect is produced so that the ions can diffuse sufficiently and the diffusion capacitance can increase rapidly. Additionally, the desorption process is a shift from high to low humidity; at high humidity, a complete layer of physically adsorbed water molecules has formed on the GO surface, but the thickness of the water molecule layer is not uniform. In areas where there are fewer water molecules, the water molecules quickly and completely release from the GO surface. As long as these areas are free of water molecules, the diffusion process is impeded, and the diffusion capacitance is rapidly reduced. Since the area where water molecules need to be released is smaller, the time is also shorter. In the adsorption process, on the other hand, a much larger adsorption area is required to form a complete chemisorption layer on the GO surface. The adsorption time is also much longer.

As we all know, humidity hysteresis is a key parameter for humidity sensor performance. The black and red curves in Figure 8b represent the water molecule adsorption and desorption curves from 10% to 90% RH, respectively. Maximum hysteresis occurs around 80%, about 1.2%. Furthermore, we measured the capacitance of the LIG_150_-60 sensor weekly for a period of 42 days to evaluate its long-term stability. The capacitance varies very little at each humidity level, which confirms that the sensor has long-term stability, as shown in Figure 8c.

#### 3.2.4. Comparison of Capacitive Humidity Sensors

Table 2 lists the different types of capacitive humidity sensors that have been recently reported, and our sensor is the most sensitive in terms of sensitivity. However, its response time is longer. From the sensor structure, the proportion of the IDE structure is larger.

### 3.3. Respiratory and Skin Humidity Monitoring

**Figure 9:**
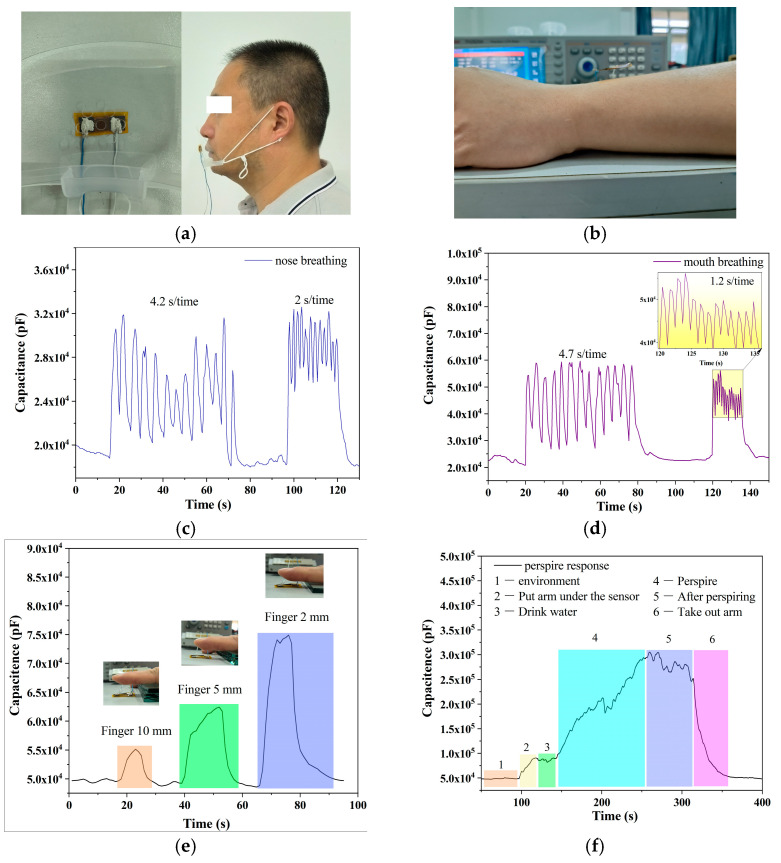
(**a**) Photograph of respiratory monitoring. (**b**) Photograph of skin humidity monitoring. The capacitance response of different breathing patterns, (**c**) nose breathing, and (**d**) mouth breathing. (**e**) Capacitance response of fingers close to the sensor at different distances. (**f**) Humidity monitoring before and after wrist perspiring.

Due to the sensor’s superior performance, it can be used for non-contact monitoring of human physiological signals, such as sweating, breathing, and non-contact fingertip techniques. Non-contact sensing is better than contact sense because it prevents sweat from contaminating the sensing surface and allows the sensor to be used repeatedly. Figure 9a is a mask for respiratory monitoring and how the mask is worn. Figure 9b is a photo that monitors sweating on the wrist. Before testing, the sensor needs to be adjusted to a height of 6 cm from the desktop. Then, put your wrist between the desktop and the sensor and keep your wrist motionless during the test. The distance may have an error of 1–2 mm, but it does not affect the trend of the detection curve. Figure 9c depicts nose breathing, while Figure 9d depicts mouth breathing. The nose breathing sensor has a variation of only a dozen nanofarads, while the mouth breathing sensor has a variation of tens of nanofarads, which is a clear difference. Compared with the oral cavity, the nasal cavity is smaller, the water content is less, and the moisture of the exhaled air is lower. Figure 9e shows the capacitance response of the finger after approaching the sensor. As can be seen, a sensor’s response varies depending on the proximity distance. For example, a capacitive sensor’s response is larger at a proximity distance of 2 mm than it is at a proximity distance of 10 mm. This characteristic has potential applications in non-contact positioning and human–computer interaction.

Figure 9f shows the non-contact monitoring of human sweating. Stage 1 represents normal ambient humidity conditions, while stage 2 is the case when the wrist is near the sensor (no contact) and the sensor capacitance changes to about 100 nF. Stage 3 represents the stage when the person drinks water, and the sensor capacitance remains basically unchanged. The fourth stage is when the human body starts to sweat, and the sensor capacitance increases rapidly. The fifth stage is when sweating reaches its peak and the sensor capacitance reaches about 250 nF. The sixth stage is when the wrist leaves. As can be seen, the sensor has a high sensitivity to human body sweat, demonstrating the potential of the sensor for monitoring human physiological processes.

## 4. Conclusions

In this work, we ablated the PI film by a picosecond laser to obtain an interdigital electrode and enhanced the humidity response by GO. This method has a simple fabrication process and lower cost. After the PI film is ablated by laser, graphene is generated on the electrode of the PI-based sensor, which contributes to the conductivity, and the smaller the electrode gap size, the greater the capacitive response. The effects of the electrode gap size and the amount of GO on the performance of GO-based sensors are discussed. The result shows that the sensitivity of the sensor is inversely related to the electrode gap size when the amount of GO is low, and when the amount of GO is large, the sensitivity is proportionate to the electrode gap. Meanwhile, there is an optimal value of the amount of GO, and the sensor is the most sensitive when the drop-coated GO is in the range of 1.45–1.86 μL/mm^2^. Due to the sensor’s high sensitivity, rapid response time, and minimal hysteresis, it can monitor human physiological signs, such as breathing and perspiration.

## Figures and Tables

**Table 1:** Sensitivity and amount of graphene oxide (GO) per unit area for different parameter sensors.

Gap Size (Electrode Area)	GO (30 μL)	GO (60 μL)	GO (90 μL)	GO (120 μL)	Max Response
50 μm(A ^1^ = 32.34 mm^2^)	0.93 μL/mm^2^ (1934 pF/RH)	1.86 μL/mm^2^ (2228 pF/RH)	2.79 μL/mm^2^ (1594 pF/RH)	3.72μL/mm^2^ (1696 pF/RH)	1.86 μL/mm^2^
150 μm(A = 41.34 mm^2^)	0.73 μL/mm^2^ (2398 pF/RH)	1.45 μL/mm^2^ (3862 pF/RH)	2.2 μL/mm^2^ (2385 pF/RH)	2.9 μL/mm^2^ (3087 pF/RH)	1.45 μL/mm^2^
350 μm(A = 59.34 mm^2^)	0.5 μL/mm^2^ (256 pF/RH)	1.01 μL/mm^2^ (2730 pF/RH)	1.51 μL/mm^2^ (3748 pF/RH)	2.02 μL/mm^2^ (3656 pF/RH)	1.51 μL/mm^2^

^1^ A denotes the area of the interdigital electrode.

**Table 2:** Comparison of recently reported capacitive humidity sensors.

Material	Structure	Frequency (Hz)	Range (%RH)	Sensitivity (pF/RH)	Response/Recovery Time (s)	Ref.
HNTs-NH_2_/PI	MIM ^a^	-	10–90	0.87	12/8	[49]
In_2_O_3_/GO	IDE ^b^	100	11–97	1061.6	15/2.5	[29]
LIG/GO	IDE	50	10–90	3215	15.8/-	[50]
PMDA/ODA/TiO_2_	MIM	1000	10–90	1.24	25/25	[51]
ZnO NR/WS_2_	Electrode	1000	18–85	0.107	74.5/25.6	[52]
GO/PDDA	IDE	10^4^	11–97	1552.3	-/-	[53]
P(VDF-TrFE)	Arc-shaped hollow	10^6^	20–90	~0.009	3.7/3.4	[54]
PCFGOM	paper cellulose	1000	10–90	0.74	1.3/0.8	[24]
Paper by laser ablation	IDE	1000	0–90	2	266/126	[55]
Ag colloidal ink	IDE	10^5^	30–85	2	250/175	[56]
GO/Nafion/In_2_O_3_	IDE	100	11–97	3080	-/-	[57]
LIG/GO	IDE	100	10–90	3862	58/15	This work

^a^ MIM: metal-insulator-metal. ^b^ IDE: interdigitated electrode.

## Data Availability

The data presented in this study are available on request from the corresponding author.
